# *Bifidobacterium longum* SX-1326 ameliorates gastrointestinal toxicity after irinotecan chemotherapy via modulating the P53 signaling pathway and brain-gut axis

**DOI:** 10.1186/s12866-023-03152-w

**Published:** 2024-01-03

**Authors:** Fenfang Yue, Xiangdi Zeng, Yufan Wang, Yilin Fang, Mengyun Yue, Xuanqi Zhao, Ruizhe Zhu, Qingwei Zeng, Jing Wei, Tingtao Chen

**Affiliations:** 1 School of Life Science, Nanchang University, Nanchang, 330031 China; 2 National Engineering Research Center for Bioengineering Drugs and the Technologies, Institute of Translational Medicine, JiangXi Medical College, Nanchang University, Nanchang, 330031 China; 3 Department of Neurology, The First Affiliated Hospital, Jiang Xi Medical College, Nanchang University, Nanchang, 330031 China

**Keywords:** Colorectal cancer, Irinotecan, Intestinal microbiota, Gastrointestinal toxicity

## Abstract

**Background:**

Colorectal cancer (CRC) is a prevalent malignant malignancy affecting the gastrointestinal tract that is usually treated clinically with chemotherapeutic agents, whereas chemotherapeutic agents can cause severe gastrointestinal toxicity, which brings great pain to patients. Therefore, finding effective adjuvant agents for chemotherapy is crucial.

**Methods:**

In this study, a CRC mouse model was successfully constructed using AOM/DSS, and the treatment was carried out by probiotic *Bifidobacterium longum* SX-1326 (*B. longum* SX-1326) in combination with irinotecan. Combining with various techniques of modern biomedical research, such as Hematoxylin and Eosin (H&E), Immunohistochemistry (IHC), Western blotting and 16S rDNA sequencing, we intend to elucidate the effect and mechanism of *B. longum* SX-1326 in improving the anticancer efficacy and reducing the side effects on the different levels of molecules, animals, and bacteria.

**Results:**

Our results showed that *B. longum* SX-1326 enhanced the expression of Cleaved Caspase-3 (M vs. U = *p* < 0.01) and down-regulated the expression level of B-cell lymphoma-2 (Bcl-2) through up-regulation of the p53 signaling pathway in CRC mice, which resulted in an adjuvant effect on the treatment of CRC with irinotecan. Moreover, *B. longum* SX-1326 was also able to regulate the gut-brain-axis (GBA) by restoring damaged enterochromaffin cells, reducing the release of 5-hydroxytryptamine (5-HT) in brain tissue (I vs. U = 89.26 vs. 75.03, *p* < 0.05), and further alleviating the adverse effects of nausea and vomiting. In addition, *B. longum* SX-1326 reversed dysbiosis in CRC model mice by increasing the levels of *Dehalobacterium*, *Ruminnococcus*, and *Mucispirillum*. And further alleviated colorectal inflammation by downregulating the TLR4/MyD88/NF-κB signaling pathway.

**Conclusions:**

In conclusion, our work reveals that *B. longum* SX-1326 has a favorable effect in adjuvant irinotecan for CRC and amelioration of post-chemotherapy side effects, and also provides the theoretical basis and data for finding a safe and efficient chemotherapeutic adjuvant.

**Supplementary Information:**

The online version contains supplementary material available at 10.1186/s12866-023-03152-w.

## Introduction

Colorectal cancer (CRC) is the most prevailing malignant tumor of the gastrointestinal tract, with incidence of more than 1.9 million new cases and approximately 900,000 deaths worldwide in 2020 [1, 2]. The global burden exerted by of CRC is projected to increase by 60% to over 2.2 million new cases and 1.1 million deaths by 2030, enabling it one of the leading cancer types threatening the health of the population [3]. Early symptoms of CRC are normally asymptomatic, as the tumor increases in size, symptoms such as hematochezia, diarrhea, and local abdominal pain are manifested, while systemic symptoms such as anemia and loss of body mass are manifested in the late stage [4]. Currently, major recommended therapeutic approach for CRC are surgery, radiotherapy and immune-targeted therapy, among which drawbacks including recurrence after surgery and toxic adverse effects of metastatic chemotherapy still remain unfixed [5]. Therefore, chemotherapy adjuvant surgery is commonly used in clinical practice to achieve better treatment results.

Irinotecan, a chemotherapeutic agent for CRC, is a topoisomerase I inhibitor that targets healthy gastrointestinal (GI) tissue and interrupts DNA synthesis, leading to apoptotic cell death [6]. Though with high therapeutic efficacy, the ongoing injury and loss of the capacity of the intestinal epithelium to rapidly self-repair exerted by irinotecan, can induce a strong inflammatory response that can elicit a range of toxic effects such as gastrointestinal toxicity (nausea, vomiting, and diarrhea) and often accompanied with dysbacteriosis which can bring huge inconvenience to patients [7]. Germ-free mice injected with irinotecan have been demonstrated can induce systemic and intestinal toxicity with simultaneous dysbacteriosis in intestinal microbiota and can lead to diarrhea at doses of 80 and 100 mg [8]. Irinotecan also causes more serious toxic side effects, including electrolyte imbalance and dehydration. As a result, the dosage and frequency of the drug are limited, and the therapeutic effect is reduced, affecting the patient's prognosis [9]. Therefore, this is an imminent need to develop ideal chemotherapeutic adjuvants which can favorably to maintain or even improve the anti-cancer efficacy of irinotecan while suppressing the side effects correlated with it.

In recent years, research on the taxa structure of intestinal microbiota and chemotherapy-induced intestinal inflammation has become a research hotspot. Intestinal microbiota is now increasingly applied to control cancer-related adverse effects and reduce regimen-related toxicity [10]. Among them, *Bifidobacterium longum* (*B. longum*), as one of the initial microorganisms colonize at host intestinal tract and also the predominant taxa of human gastrointestinal tract, exhibits an instrumental role in maintaining normal GI microenvironment [11]. Mounting animal trails and clinical studies have demonstrated that *B. longum* can alleviate colitis symptoms and further reduce chronic inflammation [12, 13]. Oral supplement of *B. longum* 51A has been reported to be effectively protect mice from irinotecan-induced intestinal damage [14]. Furthermore, it also has been reported that *B.* DD98 (*Se-B. longum* DD98) can mitigate the strong intestinal toxicity which is often associated with irinotecan therapy by reducing the expression level of pro-inflammatory cytokines IL-1β, whereas enhancing the expression of the tight junction proteins such as Occludin and further possessing the capacity to reestablish the main composition and microbial diversity of the GI microbiota [15]. Moreover, long-term ingestion of *B. longum* has been proven can prevent CRC in certain extent [16]. Our previous work also showed that *B. longum* SX-1326 could exert anti-aging effects by modulating the gut microbiota in combination with several probiotics to inhibit TLR4/NFκB-induced intestinal inflammation and increase the expression of the intestinal permeability-associated proteins occlusion zone-1 (ZO-1).

In the present study, we investigated the effects of *Bifidobacterium longum* SX-1326 (*B. longum* SX-1326) on CRC which was extracted from feces of centenarians. Firstly, CRC mice model was established by AOM/DSS, and the therapeutic efficacy and potential molecular mechanism of *B. longum* SX-1326 combined with irinotecan for CRC were investigated by Immunohistochemistry (IHC) and Western blotting. Moreover, the efficacy of *B. longum* SX-1326 alleviating diarrhea and vomiting after irinotecan chemotherapy was further explored by analyzing disease activity index score, Hematoxylin and Eosin (H&E) staining and enzyme-linked immunosorbent assay (ELISA). Our goal is to provide a new approach for the clinical treatment of CRC, and also to provide a theoretical basis and data for finding a safe and effective chemotherapy adjuvant.

## Materials and methods

### Strain screening

*B. longum* SX-1326 was isolated from the feces of seven centenarians (ages 103, 107, 102, 105, 100, 101, and 100) in Centenarian Village, Ganzhou City, Jiangxi Province, China. The study received review and approval from the Regional Ethics Committee of Zhanggong District (approval number: [2019] 001).

Fresh fecal samples were diluted with PBS to different gradients, and 30 μL of the diluted fecal solution of different concentrations was spread on MRS solid culture medium containing 0.05% (w/v) cysteine. The plates were then incubated in an incubator for 36 h (37 °C, anaerobic). Single colonies were selected based on differences in colony morphology, size, and color, and were then subcultured for purification. Extraction of genomic DNA from purified bacteria and bacterial identification using the NCBI database. *B. longum* SX-1326 was from Jiangxi Shangtai Health Industry Development Company Limited, Shangrao, Jiangxi, China and preserved in the China General Microbiological Culture Collection Center (CGMCC No.19853) for preservation [17].

### Animal and experimental design

C57BL/6 mice (6 weeks, 19–21 g) were from Hunan Slack Jingda Laboratory Animal Co, Ltd. All mice were maintained under a standard animal room (12/12 light–dark cycle, humidity between 45%-55%, temperature of 23–25 °C). Mice were given free access to sterilized water and rodent chow. In order to avoid the influence of different time periods, the animal experiments were conducted between 9:00 am and 12:30 pm.

Then all 50 mice were equally randomized into five groups: (1) C Group (*n* = 10), mice did not apply any treatment; (2) M Group (*n* = 10), each mouse was intraperitoneally injected (10.0 mg/kg) with 100 μL saline dissolved with azomethane oxide (AOM) at first day of modeling, and after drinking normal water for 7 days, 2.5% dextran sodium sulfate (DSS) was given within water for following 7 days, and then drinking normal water for next 7 days, for a total of three cycles; (3) B Group (*n* = 10), after completion of CRC-molding same as M group, gavage treatment with *B. longum* SX-1326 (10^9^ CFU/ml) was performed for consistent 3 weeks; (4) I Group (*n* = 10), after the end of molding, irinotecan was intraperitoneally injected with 100 μL (75 mg/kg) for 3 days; (5) U group (*n* = 10), after the completion of molding, irinotecan 100 μL (75 mg/kg) was injected intraperitoneally for 3 days, and subsequently 10^9^ CFU/ml of *B. longum* SX-1326 was used for gavage for next three weeks. Alternations in weight were measured weekly, and mouse fecal samples were collected before the end of treatment. After inhalation of isoflurane anesthesia, blood, colon tissue, feces, and brain tissue were collected for biochemical and histological analysis.

This study was reviewed and approved by the Laboratory Animal Welfare Ethics Committee of Nanchang Leyou Biotechnology Co. (Approval number: RYE2021090902).

### Kaolin experiment

Kaolin was mixed with 3% (w/w) gum arabic in sterilized water to make pellets similar in shape and size to the feed and dried completely (room temperature). 100 g each of normal feed and kaolin were weighed and fed ad libitum at constant temperature and under normal light. The amount of kaolin ingested by each group of mice was recorded regularly every day for 24 h after the drug intervention. Mouse xenophobic behavior was characterized according to the amount of kaolin ingested by each group and was used to assess the degree of nausea and vomiting [18, 19].

### Disease activity index score

To determine whether *B. longum* SX-1326 gavage alters the extent of disease in mice with CRC, the disease activity index was examined in each group. Specifically, changes in body weight of mice in each group were monitored at the end of modeling; feces of mice in each group were collected at the end of treatment to observe the degree of thinness and softness and the degree of occult blood, and were scored. According to the scoring criteria of Cooper HS: DAI = (fraction of weight loss + fraction of stool traits + fraction of blood in the stool): (1) stool consistency (0 = normal; 2 = very soft; 4 = liquid); (2) blood in the stool (0 = normal; 2 = positive occult blood; 4 = gross bloody stool); (3) the animal's reduction (0 = less than 1% weight reduction; 1 = weight reduction of 1 to 5%; 2 = weight reduction of 6 to 10%; 3 = weight reduction of 11 to 15%; 4 = weight reduction > 16%) [20].

### H&E staining

In order to observe the nuclear division of tumor tissues of mice in the M group and the structural integrity and inflammatory infiltration of the colorectum of mice in each group, tumor tissues of mice in the M group and colonic tissues of mice in each group were selected, fixed in paraformaldehyde solution for 24 h, dehydrated in graded alcohol solution, and then paraffin-embedded. The samples were cut to obtain ultrathin sections and then stained with H&E. Finally, tissue damage was analyzed using a pathology section scanner [21].

### RT-qPCR

To detect the transcript levels of the target genes, total RNA was extracted from the brain and colon tissues of each group of mice. After the RNA concentration is determined, it is transcribed to cDNA using the PrimeScript™ RT kit (Takara, RR047A). RT-qPCR was performed using the TB Green Premix Ex Taq II Kit (Takara, RR820A). The program was 95 °C (30 s), followed by 40 cycles of 95 °C (5 s) and then 60 °C (34 s). Primers are listed in Supplementary Material Table S1. Analyzed using the 2^−ΔΔCt^ method [22].

### Immunohistochemistry (IHC)

To observe the expression of target proteins in colon and brain tissues of mice in each group, brain and colon tissue sections fixed in 4% paraformaldehyde were deparaffinized and hydrated. The sections were incubated with 3% H_2_O_2_ for 10 min and 5% goat serum for 1 h (room temperature). The sections were finally incubated overnight (4 °C) with primary antibodies as in Supplementary Table 2. Immunohistochemical staining was completed with secondary antibody. Finally, observe and record the expression of the target protein under a microscope [23].

### Western blotting analysis

After homogenization of colon and tumor tissues, proteins were lysed using protease inhibitor RIPA lysis solution (Solarbio, China, R0010), and proteins were obtained by low-temperature centrifugation (1000 rpm, 20 min). After determining the protein concentration, gels with different concentrations were prepared according to the molecular weight size for electrophoresis. The membrane was transferred, closed, and then co-incubated with primary antibody overnight (4 °C). The membrane was washed, and the secondary antibody was incubated for 2 h with the antibody as in Supplementary Table 2. The membrane was washed and developed using chemiluminescent solution (Thermo Fisher, 32,209) [23].

### ELISA

The colorectal tissue and brain tissue samples of each group of mice were removed from the refrigerator at -80℃, fully homogenized using a homogenizer, centrifuged at 5000 rpm for 10 min, and the supernatant was taken as a backup. Levels of 5-HT in brain and colorectal tissues were measured using an ELISA kit (YI FEI XUE BIOTECHNOLOGY, YFXEM00037) according to the kit instructions [24].

### High-volume sequencing analysis

To detect the diversity of fecal flora in each group of mice, feces from each group were collected for differential analysis. Bacterial genomic DNA sequences were obtained by DNA extraction kit (DP712, Tiangen, Beijing, China). The target fragment of 16S rRNA V4 region was amplified by universal primers (515F/806R), and the amplified products were sequenced by Illumina platform (San Diego, California, USA) to obtain ASV/OUT feature sequences. Based on ASV/OUT, α-diversity, β-diversity and species difference analyses were performed [23].

### Data analysis

Data are presented as mean ± standard error and analyzed by ANOVA followed by Tukey's post hoc test. Graphs were statistically analyzed using GraphPad Prism version 9.0 (GraphPad Software, San Diego, USA) at *p* < 0.05 (*), *p* < 0.01 (**).

## Results

### Construction of a mouse model of colorectal cancer

**Fig. 1:**
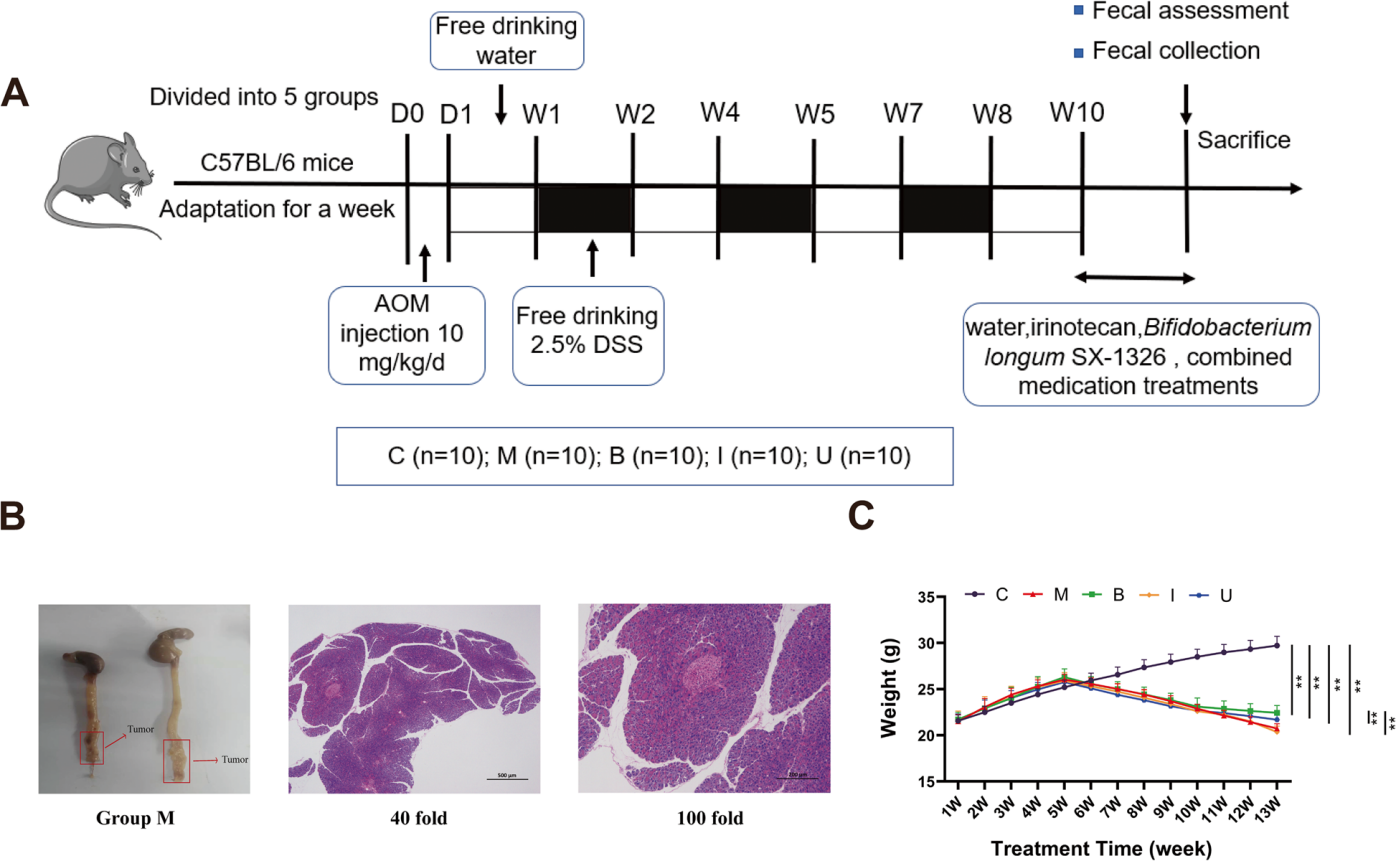
Schematic of whole experimental schedule and construction of a mouse model of CRC. **A** Processing schedule to explain experimental design of animal experiments; **B** Monitoring colorectal neoplasia and H&E staining tumor tissue; **C** Weekly body weight change of mice in each group. Experimental group: M: colorectal cancer model group; B: *Bifidobacterium longum* SX-1326 group; I: Irinotecan chemotherapy group; U: *Bifidobacterium longum* SX-1326 plus irinotecan dosing group.Data are expressed as mean ± SD, *p < 0.05, **p < 0.01

In order to evaluate the antineoplastic effect of *B. longum* SX-1326 in combination with irinotecan, we induced CRC in mice using AOM/DSS (Fig. 1A). Two mice were randomly selected for dissection, and tumor formation was seen in the colonic region. The experimental results of H&E staining indicated that the tumor part of the colon demonstrated significant changes in its pathology, including the presence of a liquefied necrosis zone in the middle area, neutrophil infiltration, and pathological nuclear division image, revealing that a mouse model of CRC was smoothly constructed (Fig. 1B). In our study, the results showed that mice in I group had a reduced body weight (*p* < 0.01) and slowing of weight loss in mice after giving *B. longum* SX-1326 gavage (*p* < 0.01) (Fig. 1C).

### Irinotecan combined with *B. longum* SX-1326 exert antineoplastic effects by promoting the p53 and Cleaved Caspase-3 apoptosis pathways.

**Fig. 2:**
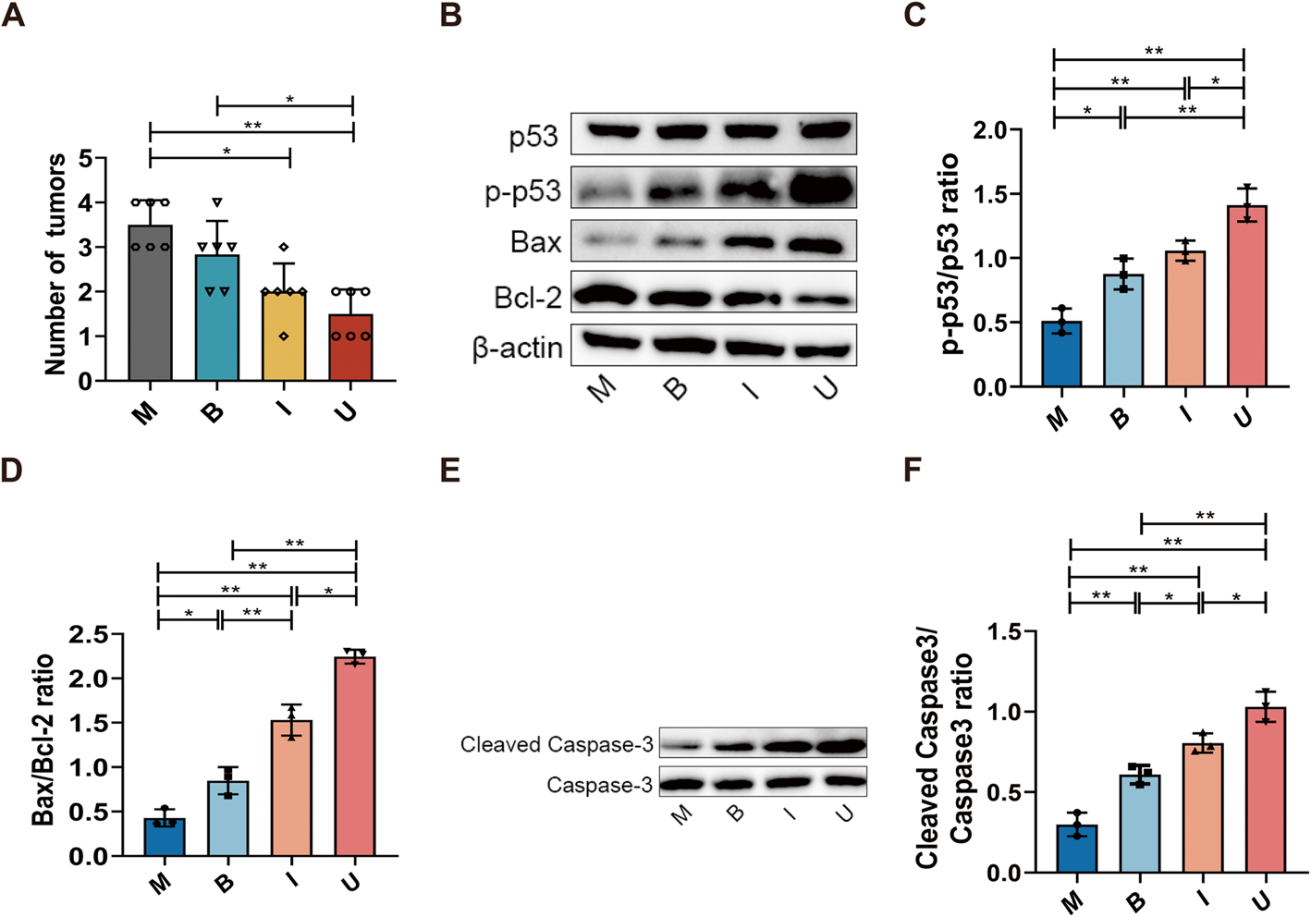
*B. longum* SX-1326 in combination with irinotecan upregulates the expression of apoptotic proteins to exert anti-tumor effects. **A** Monitor the number of mouse tumors; **B** Detection of expression of p53-induced apoptosis signaling pathway protein in tumor tissue by WB; **C**-**F** WB results of p-p53, Bax and Bcl-2 in tumor tissues were quantified using ImageJ; H. Detection of expression of Cleaved Caspase-3 in tumor tissue by WB; I. WB results of Cleaved Caspase-3 in tumor tissues were quantified using ImageJ. Experimental group: M: colorectal cancer model group; B: *Bifidobacterium longum* SX-1326 group; I: Irinotecan chemotherapy group; U: *Bifidobacterium longum* SX-1326 plus irinotecan dosing group. Data are expressed as mean ± SD, *p < 0.05, **p < 0.01

After treatment by giving a combination of *B. longum* SX-1326 and irinotecan, we found that the amount of colon tissue tumors in U group was reduced compared to M group (*p* < 0.01) **(**Fig. 2A). To further investigate the molecular mechanism of the effect of combination drugs on antineoplastic, we used Western blotting to detect the expression level of related proteins in tumor tissues. The results showed that the use of irinotecan and the combination of drugs both activated phosphorylation of p53, which initiated the DNA repair mechanism compared to M group (Fig. 2B). p53, as a transcription factor, further activated the transcription of downstream apoptotic genes, and compared with M group, the ratio of Bax/Bcl-2 in U group was significantly increased (*p* < 0.01), and the expression level of the apoptotic protein Cleaved Caspase-3 was also significantly increased (*p* < 0.01) (Fig. 2C-F). These results show that *B. longum* SX-1326 can promote apoptosis of tumor cells in combination with irinotecan and adjuvant the treatment of CRC.

### *B. longum* SX-1326 inhibit nausea and vomiting caused by chemotherapy drugs by regulating neurotransmitters in the GBA.

**Fig. 3:**
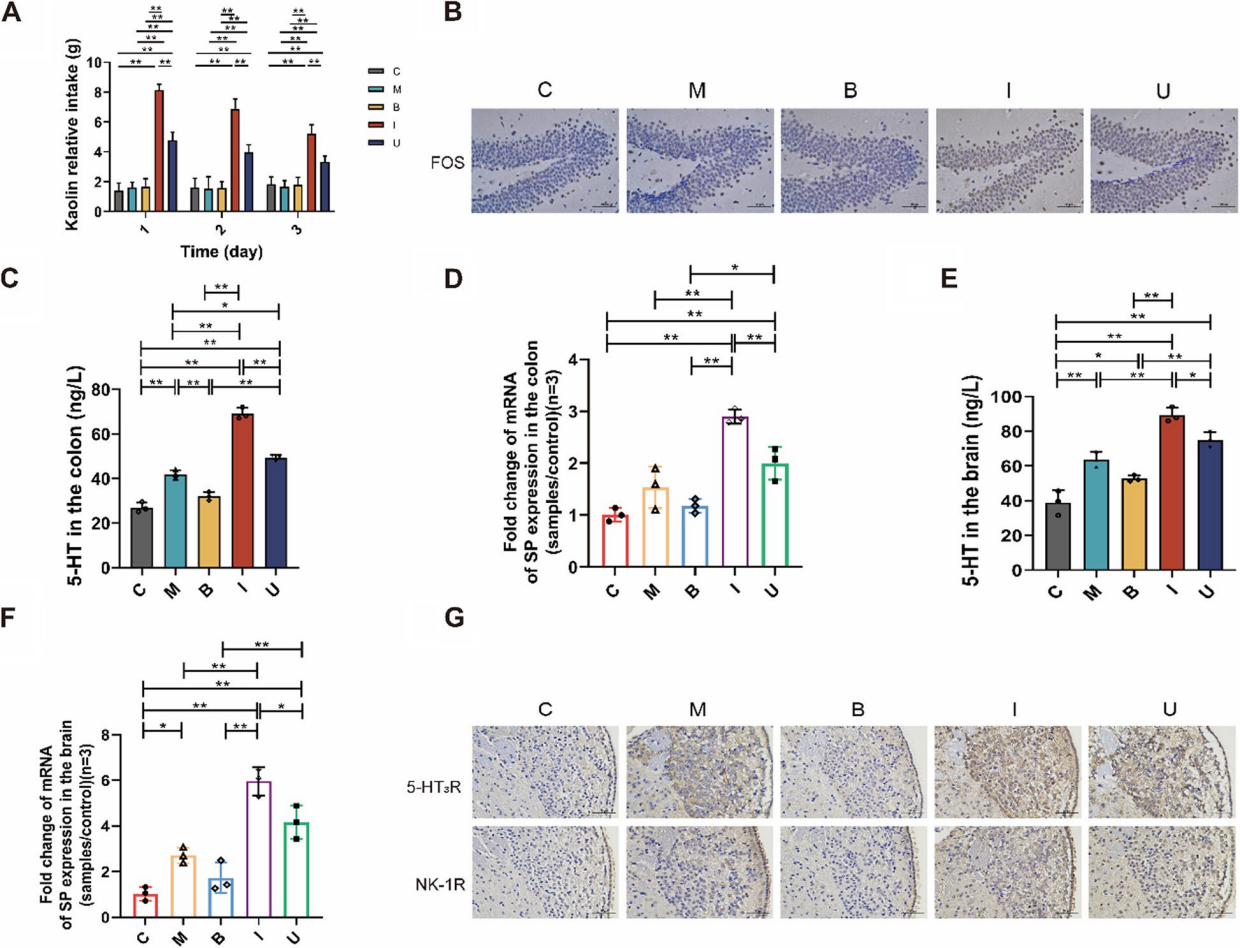
*B. longum SX-1326* inhibits nausea and vomiting caused by chemotherapy drugs by modulating neurotransmitters such as SP and 5-HT in the gut-brain axis. **A** The kaolin weight to total feed intake of mice in each group; **B** Expression of FOS protein in AP region of brain tissue by immunohistochemistry (400 ×); **C** ELISA detects the expression of 5-HT in colon tissue; **D** RT-qPCR detects SP expression in colon tissue; **E** ELISA detection of 5-HT expression in brain tissue; **F** RT-qPCR to detect SP expression in brain tissues; **G** Detection of expression of 5-HT3R and NK-1R in AP region of brain tissue by IHC staining (400 ×). Experimental group: C: normal control group; M: colorectal cancer model group; B: *Bifidobacterium longum* SX-1326 group; I: Irinotecan chemotherapy group; U: *Bifidobacterium longum* SX-1326 plus irinotecan dosing group. Data are expressed as mean ± SD, *p < 0.05, **p < 0.01

Some studies have reported that irinotecan treatment for CRC can cause adverse reactions such as vomiting. To further investigate the effect of *B. longum* SX-1326 on vomiting after irinotecan chemotherapy, a kaolin experiment was first used to verify (Fig. 3A). The results demonstrated that the kaolin intake of mice in group I was increased compared with C group (C vs. I = 1.689 vs. 6.733, *p* < 0.01). The kaolin intake of mice in the U group given *B. longum* SX-1326 after chemotherapy decreased significantly (I vs. U = 6.733 vs. 4.028, *p* < 0.01). To further explore the expression of markers of neuronal activity in mice, the c-FOS (FOS) protein in the area postrema (AP) of the mouse brain was stained by IHC (Fig. 3B). The results showed that compared with C group, the expression of FOS protein increased in mice in I group, while the expression of FOS protein decreased in U group after the combination of drugs.

To further explore the regulation of neurotransmitter expression after chemotherapy by *B. longum* SX-1326, we detected the expression of 5-hydroxytryptamine (5-HT) in the colon of each group of mice using ELISA (Fig. 3C). The results indicated that the content of 5-HT in the colon of I group was significantly increased (C vs. I = 26.98 vs. 69.07, *p* < 0.01), the U group could downregulate the expression of chemotherapy-induced 5-HT (I vs. U = 69.07 vs. 49.14, *p* < 0.01). Furthermore, the detection of Substance P (SP) in the gut of mice in each group using RT-qPCR (Fig. 3D), the expression of SP in I group increased (*p* < 0.01) and decreased its expression in U group (*p* < 0.01). Preclinical evidence has firmly established bidirectional interactions between the gut and the brain. For this reason, ELISA was used to quantitatively detect the expression of the neurotransmitter 5-HT in the brains of mice in each group, and compared with C group, the expression level of 5-HT in I group was significantly increased (C vs. I = 38.87 vs. 89.26, *p* < 0.01), whereas the expression in U group decreased (I vs. U = 89.26 vs. 75.03, *p* < 0.05) (Fig. 3E). In addition, SP was detected in mice in each group using RT-qPCR, and consistently, expression was significantly increased in I group (*p* < 0.01), and U group was able to reverse this trend (*p* < 0.05) (Fig. 3F). IHC detection of the relevant neurotransmitter receptors 5-HT_3_R and NK-1R in the AP region of the mouse brain (Fig. 3G) showed that expression was increased in I group compared to C group, and this trend was reduced after combined administration. These results show that *B. longum* SX-1326 can regulate the expression of neurotransmitters 5-HT and SP through the gut-brain-axis (GBA), inhibit the activation of the vomiting center by the central nervous system, thereby reducing the occurrence of vomiting.

### *B. longum* SX-1326 can reverse the dysbacteriosis of intestinal microbiota caused by irinotecan chemotherapy.

**Fig. 4:**
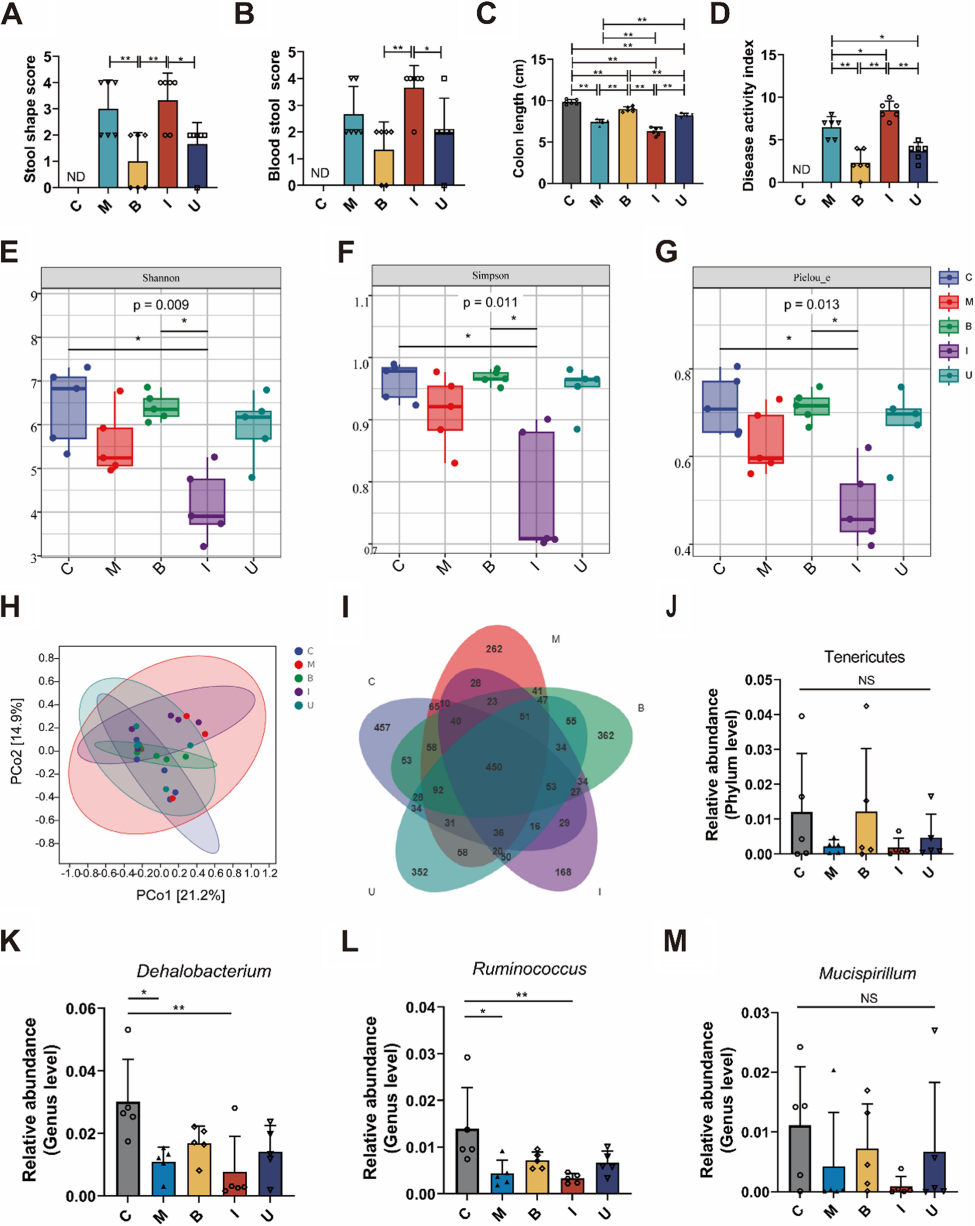
*B. longum SX-1326* balances dysregulated gut microbiota caused by chemotherapy. **A** Fecal shape scoring of mice in each group; **B** Blood stool scoring of mice in each group; **C** Colorectal length of mice in each group; **D** Disease activity index scores for each group of mice (**E**) Shannon index; **F** Simspon index; **G** Pielou_e analysis; **H** PCoA diagram; **I** Venn diagram; **J** Tenericutes; **K** *Dehalobacterium*; **L** *Ruminnococcus*; **M** *Mucispirillum*. Experimental group: C: normal control group; M: colorectal cancer model group; B: *Bifidobacterium longum* SX-1326 group; I: Irinotecan chemotherapy group; U: *Bifidobacterium longum* SX-1326 plus irinotecan dosing group. Data are expressed as mean ± SD, *p < 0.05, **p < 0.01

To evaluate the therapeutic effect of *B. longum* SX-1326 on the intestinal microbiota after chemotherapy, the feces of each group of mice were first scored (Fig. 4A-B), and the study showed that both M and I groups mice developed loose, thin, bloody stools. Compared to I group, mice in both B group (*p* < 0.01) and U group (*p* < 0.05) had a lower probability of blood stool, while B group had a better effect. Measuring colorectal length in each group of mice showed that (Fig. 4C), colorectal length in I group (C vs. I = 9.833 vs. 6.300, *p* < 0.01) was significantly shorter than in C group, while colorectal length was restored by administration of *B. longum* SX-1326 (B vs. I = 8.950 vs. 6.300, *p* < 0.01) and combined administration (I vs. U = 6.300 vs. 8.233, *p* < 0.01). In addition, the DAI of mice in the U group was reduced compared to I group (*p* < 0.01) (Fig. 4D).

In addition, relevant studies have suggested that intestinal microbiota disorders are closely related to the development and progression of CRC, and we used 16S rDNA high-throughput sequencing to analyze the feces of mice. Shannon, Simpson and Pielou_e indices were used to assess the α-diversity of the intestinal microbiota of mice (Fig. 4E-G). Compared with C group, the α-diversity of gut microbes decreased in I group (*p* < 0.05), while B group was able to effectively reverse the decrease in α diversity (*p* < 0.05). The β-diversity of the gut microbial taxa of mice in each group was further evaluated by PCoA principal component analysis, and the β diversity of the gut microbiota of the I group mice was altered compared to C group, while this change was reversed after gavage of *B. longum* SX-1326 (Fig. 4H). Venn demonstrated that the intestinal microbiota of mice in C, M, B, I and U groups had a total of 450 identical OTUs, and the unique OTUs were 457, 262, 362, 168, and 352 respectively (Fig. 4I). At the phylum level, the abundance of Tenericutes in B group was elevated compared to and I group, though there was no statistical difference (Fig. 4J). At the genus level, we selected some microbiota associated with CRC for analysis, with reduced relative abundances of *Dehalobacterium* (Fig. 4K) and *Ruminnococcus* (Fig. 4L) in I Group comparing with C Group (*p* < 0.01). The results showed that chemotherapy in CRC mice reduced the relative abundance of *Mucispirillum* (Fig. 4M), and treatment with *B. longum* SX-1326 was able to reverse this trend. The above results show that treatment with *B. longum* SX-1326 can reverse the dysregulation of intestinal microbiota after chemotherapy in mouse models of CRC and restore the diversity of microbiota.

### *B. longum* SX-1326 can improve intestinal inflammation in CRC mice and relieve diarrhea caused by chemotherapy drugs.

**Fig. 5:**
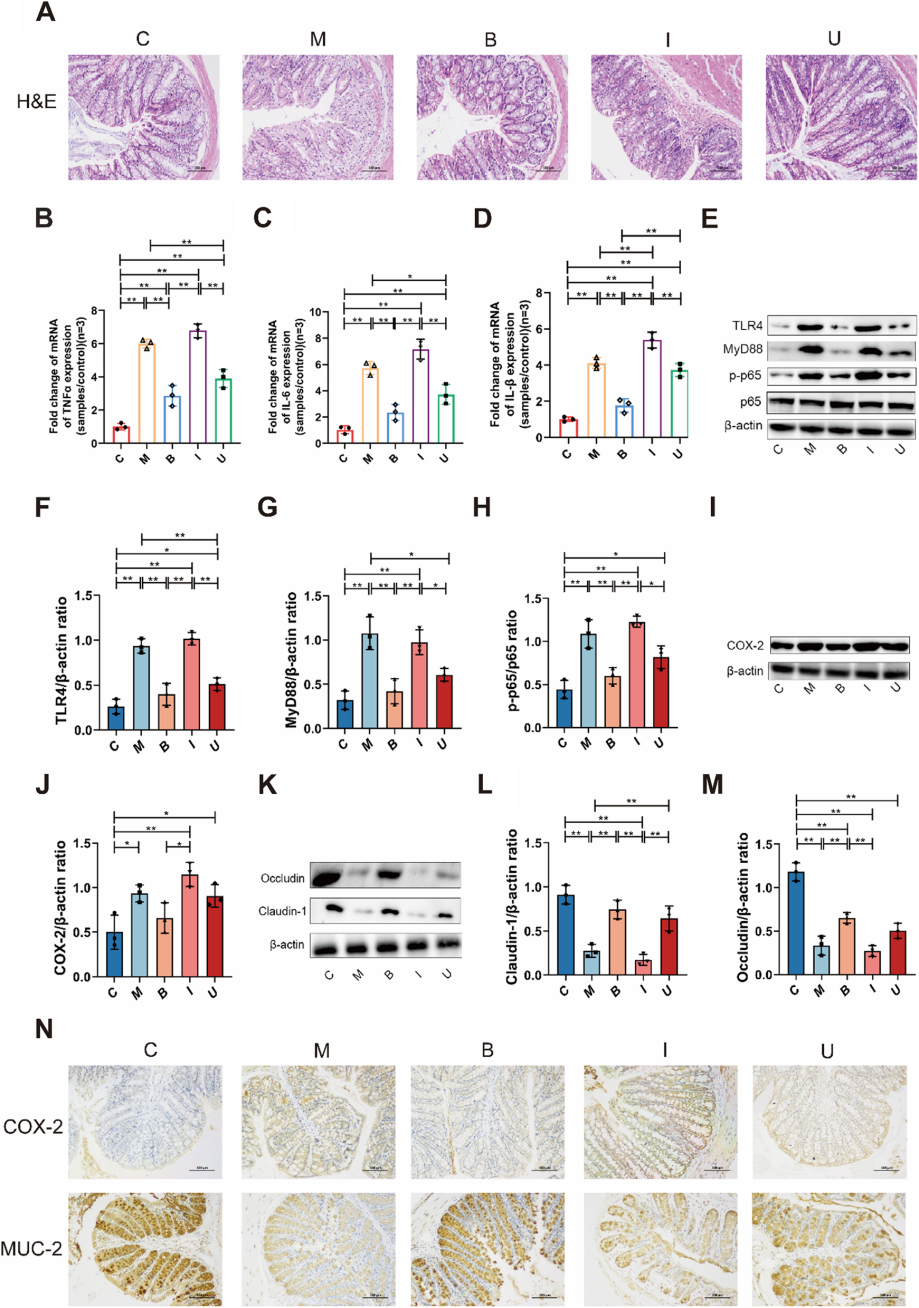
*B. longum SX-1326* improves intestinal inflammation in CRC and relieves diarrhea caused by chemotherapy alone. **A** H&E staining to observe colonic inflammatory cell infiltration and morphological changes (200 ×); **B**-**D** RT-qPCR detected the expression of inflammatory factors TNF-α, IL-6 and IL-1β in the intestines of mice in each group. **E** WB detection of expression of TLR4-MyD88-p65-p-p65 signaling pathway protein in the gut; **F**–**H** WB results for TLR4, MyD88, p65 and p-p65 were quantified using ImageJ; **I** WB detection of intestinal COX-2 expression; **J** Quantitative analysis of WB results for COX-2 using ImageJ; **K** WB detection of expression of barrier proteins Claudin-1 and Occludin in the gut; **L**-**M** WB results for Claudin-1 and Occludin were quantified using ImageJ; **N** COX-2 and MUC-2 expression in colorectal tissues was detected by immunohistochemical staining (200 ×). Experimental group: C: normal control group; M: colorectal cancer model group; B: *Bifidobacterium longum* SX-1326 group; I: Irinotecan chemotherapy group; U: *Bifidobacterium longum* SX-1326 plus irinotecan dosing group. Data are expressed as mean ± SD, *p < 0.05, **p < 0.01

To further clarify the effect of *B. longum* SX-1326 on intestinal inflammation in CRC mice after chemotherapy, H&E staining was performed on the colons of each group of mice (Fig. 5A). Compared with C, pathological phenomena such as inflammatory cell infiltration occurred in both M and I group, while intestinal inflammatory cell infiltration decreased and colonic structure returned to normal in B and U groups, among which the improvement effect was particularly obvious in B group. The results of RT-qPCR detection of colorectal tissue-associated inflammatory factors (Fig. 5B-D) showed that tumor necrosis factor (TNF-α), interleukin-6 (IL-6), and interleukin-1β (IL-1β) were significantly elevated in I group (*p* < 0.01) compared to C group. The combination (*p* < 0.01) inhibited the expression of the above inflammatory factors after chemotherapy, and we also detected the changes of intestinal inflammation-related proteins such as p65 through Western blotting (Fig. 5E). The results indicated that the expression of TLR4, MyD88 and p-p65 was reduced in both B and U groups compared with group I, and more interestingly, the effect of inhibiting the expression of the above inflammatory proteins was more significant when B group was used (*p* < 0.01) (Fig. 5F-H). Moreover, the results of Western blotting for COX-2 showed that expression was reduced in B group compared with I group (*p* < 0.05) (Fig. 5I-J).

However, intestinal inflammation and barrier disruption often accompany it, so we also tested the intestinal barrier functional protein by Western blotting (Fig. 5K), and compared with C group, Claudin-1 and Occludin expression was reduced in I group (*p* < 0.01), and Claudin-1 restored in U group (*p* < 0.01) (Fig. 5L-M). The results of IHC detection of intestinal mucosal protein (MUC-2) demonstrated that the expression of MUC-2 protein in I group was reduced compared with C group, while the increase of MUC-2 protein in U group (Fig. 5N). In conclusion, treatment with *B. longum* SX-1326 can inhibit the expression of related inflammatory proteins, thereby reducing intestinal inflammation, while increasing the expression of mucin and restoring intestinal barrier function, thereby alleviating diarrhea caused by chemotherapy.

## Discussion

CRC is one of the most prevalent typology of cancer in humans, has high morbidity and mortality rates [25]. Irinotecan is used as a first-line drug for CRC, and although it has some efficacy, this chemotherapy can lead to nausea and vomiting; mucosal barrier dysfunction [26]. Therefore, the search for an adjuvant that maintains or even improves the anticancer efficacy of irinotecan and also reduces side effects is necessary.

Here, we evaluated the antitumor effect of *B. longum* SX-1326 by gavage using AOM/DSS-induced CRC mouse model. Our data showed that *B. longum* SX-1326 in combination with irinotecan reversed chemotherapy-induced weight loss and inhibited CRC development and progression in mice. Previous studies have shown that the active metabolite SN-38 can bind with the topoisomerase I-DNA complex during chemotherapy with irinotecan and affects the recombination of DNA strands, which in turn leads to DNA double-strand breaks and genetic damage [27]. Sustained damage accumulates pro-apoptotic p53 target genes when the cell cycle is arrested and DNA is irreparable. p53 as a transcription factor can further activate the transcription of downstream pro-apoptotic genes, and the two major pathways trigger apoptosis [28, 29]. Mitochondrial membrane permeabilization (MOMP) activates endogenous pathways and receptor signaling triggers exogenous pathways [30]. In studying the interaction of p53 with Bcl2 proteins, acetylation of the p53 DNA-binding domain promotes interaction with pro-apoptotic proteins (Bax) and Bcl2 antagonist/killer (BAK). Meanwhile, when Caspase-3 is activated, a cascade reaction of apoptotic proteases occurs [31, 32]. The activated enzyme degrades important intracellular proteins, ultimately leading to irreversible apoptosis [33]. When gavaged, *B. longum* SX-1326enhances the expression of Cleaved Caspase3, an apoptosis-regulating gene in CRC, while down-regulating the expression of Bcl-2, thus achieving adjuvant irinotecan for the treatment of CRC (Figs. 1 and 2).

Nausea and vomiting are two of the most disturbing side-effects present during and after CRC chemotherapeutic, and are statistically experienced by 3/4 of patients [34, 35]. Irinotecan chemotherapy has been reported to cause damage to enterocytes and increased levels of 5-HT secreted by intestinal chromaffin cells [36]. 5-HT can be synthesized by the tryptophan hydroxylases (TpH1, TpH2) in intestinal and neuronal enterochromaffin cells, respectively, and accounts for approximately 95% of the neurotransmitters in the gut, which have a role in controlling mood, sleep, inflammation, and intestinal barrier functions [37]. 5-HT signaling is dependent on specific receptor subtypes and is terminated by serotonin transporter (SERT) [38]. Clinical studies have shown that the gut and the central nervous system (CNS) can be closely linked through bidirectional communication via the GBA. The bidirectional communication network of the GBA also includes the autonomic nervous system and the hypothalamic–pituitary–adrenal axis [39]. Intestinal communication to the central nervous system is autonomous, but under pathological conditions, signals are transmitted to the sensory system, leading to symptoms such as nausea. Similarly, the central nervous system can act in reverse to cause gastrointestinal dysfunction [40]. Thus released 5-HT may agonize the 5-HT3R located on vagal nerve endings in the intestinal wall, affecting the vomiting center and thus leading to vomiting [24]. We examined the expressivity of 5-HT and SP in colorectal and brain tissues using ELISA and RT-qPCR, and found that the expression of both was reduced in both intestinal and brain tissues after *B. longum* SX-1326 intervention. It was confirmed that *B. longum* could promote the secretion of mucus from epithelial cells to make them form a mucus layer, improve the mucosal defense ability, and alleviate the damage of intestinal chromaffin cells and intestinal epithelial cells caused by chemotherapy [41, 42]. It was shown that *B. longum* SX-1326 could alleviate nausea and vomiting by regulating the GBA and decreasing the expression of neurotransmitters in brain tissue (Fig. 3).

In addition, many studies have shown that irinotecan also causes severe dysbiosis of the intestinal flora, leading to a decrease in flora diversity [43, 44]. To assess the effect of *B. longum* SX-1326 on intestinal microecology, we first observed the feces of each group of mice scoring. *B. longum* SX-1326 combined with irinotecan treatment was found to alleviate dilute and bloody stools in mice. High-throughput sequencing analysis showed that *B. longum* SX-1326 restored the α-diversity and β-diversity of the intestinal microbiota in CRC mice. Venn diagrams also indicated that *B. longum* SX-1326 restored bacterial diversity to some extent. In the present study, several bacteria showed increased abundance at the genus level, including *Dehalobacterium* and *Ruminnococcus*. One study found that *Dehalobacterium*, as an intestinal probiotic, was able to promote butyric acid production [45]. *Ruminnococcus*, as an intestinal cornerstone bacterium, has been associated with intestinal disorders, stabilizing the intestinal barrier, reversing diarrhea, and reduce CRC risk [46]. *Mucispirillum* abundance decreases during CRC disease progression. It has been shown to fight *Salmonella* and resist intestinal inflammation, and treatment with *B. longum* SX-1326 restores this dysbiosis [47] (Fig. 4).

**Fig. 6:**
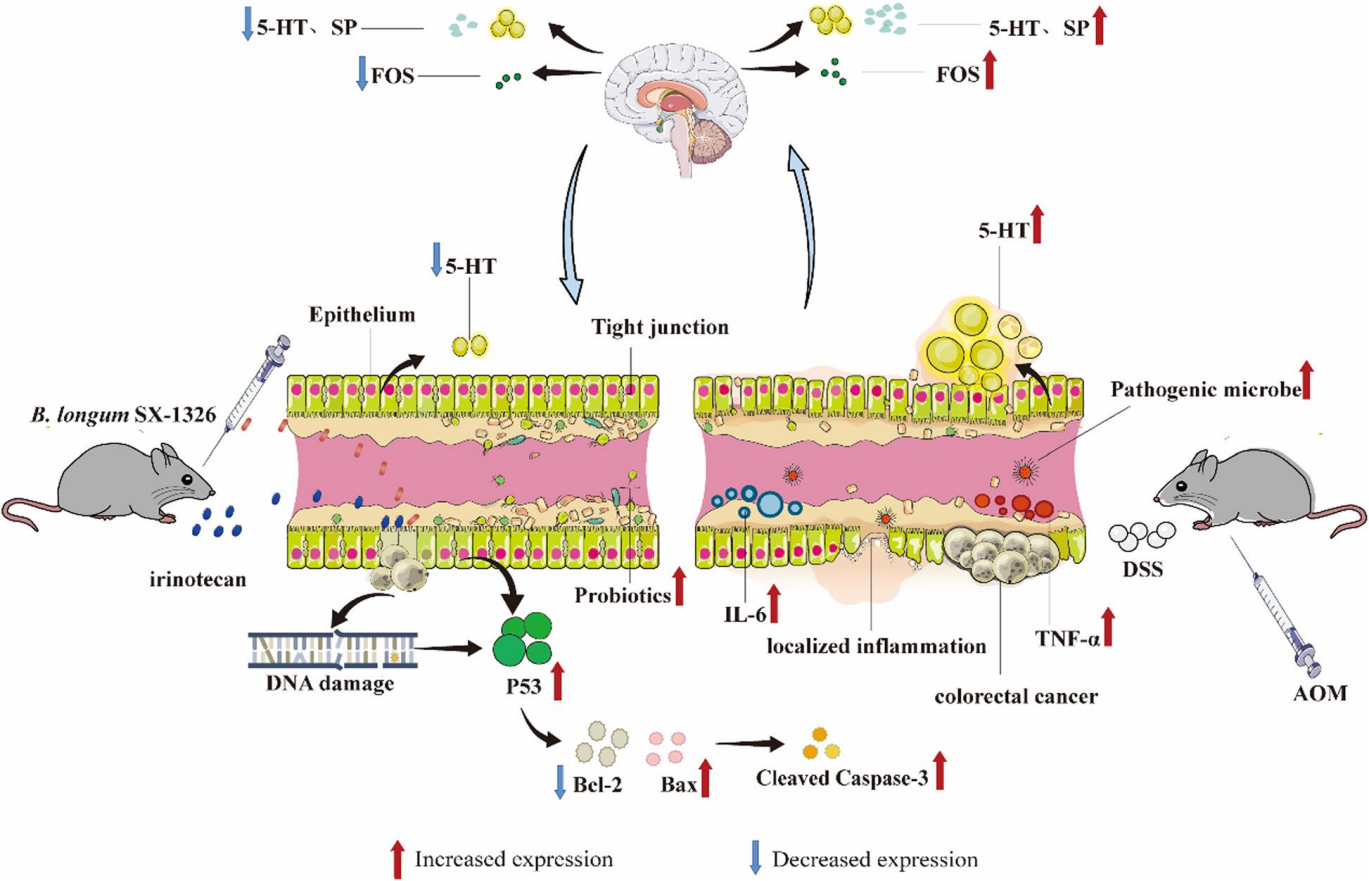
Diagram of the potential mechanism of *B. longum* SX-1326 for adjuvant treatment of CRC and amelioration of post-chemotherapy side effects. In AOM and DSS-induced CRC mouse models, *B. longum* SX-1326 was able to enhance the expression of pro-apoptotic proteins to assist in the treatment of CRC, regulate the release of neurotransmitters, such as 5-HT, through the GBA to improve nausea and vomiting, and restore the diversity of the intestinal flora to alleviate intestinal inflammation. AOM, Azoxymethane; DSS, Dextran Sulfate Sodium Salt

Histopathologic analysis indicated that irinotecan chemotherapy can cause intestinal tissue damage, including structural loss and neutrophil infiltrate [48]. Inflammation of intestinal tissue is induced when pro-inflammatory cytokines are increased in the intestinal mucosa. Activated immune cells receive signals and secrete large amounts of inflammatory cytokines, including TNF-α, IL-1β and IL-6 [49]. Clinical trials and animal studies have shown that *B. longum* prevents the exacerbation of colitis and relieves its clinical symptoms [50, 51]. It has been shown that *B. longum* reduces the incidence of spontaneous and chemically induced colitis by remodulating cytokines level or stimulating immunomodulatory mechanisms in a specific manner. In addition, intestinal epithelial cells and various immune cells form another defense barrier that regulates the immune system through the secretion of various cytokines, thereby resisting the invasion of pathogenic microorganisms [52, 53]. For instance, TNF-α secreted by Th1 cells induces a variety of pro-inflammatory responses, and Th17 cells activate and recruit neutrophils [54]. In line with this, we demonstrated by RT-qPCR experiments that gavage of *B. longum* SX-1326 decline the expression of TNF-α, IL-6 and IL-1β in colorectal tissues. Western blotting experiments further confirmed that treatment with *B. longum* SX-1326 down-regulated the expression level of key proteins related to the TLR4/MyD88/NF-κB signaling pathway. In addition, Schroeder et al. found that *B. longum* increased mucus secretion, restored the intestinal mucus layer, and decreased intestinal permeability [55]. Concordant with this, our results also showed that *B. longum* SX-1326 up-regulated the expression of Occludin and Claudin-1 and restored the intestinal barrier function. Previous studies have suggested that chemotherapy utilizing irinotecan increases the expression of COX-2, which subsequently causes an increase in the level of PGE2, thereby causing diarrhea by stimulating colonic secretion and peristalsis [56]. For this reason, we demonstrated the ability of *B. longum* SX-1326 to reduce COX-2 expression in colonic tissues after treatment by Western blotting and IHC. Our results suggest that *B. longum* SX-1326 can alleviate chemotherapy-induced diarrhea by improving intestinal inflammation and restoring intestinal barrier function (Fig. 5). In conclusion, *B. longum* SX-1326 can effectively assist irinotecan in the treatment of CRC by enhancing pro-apoptotic proteins; regulate the GBA to reduce the expression of neurotransmitters to alleviate nausea and vomiting after chemotherapy; and restore the diversity of the intestinal flora to reduce intestinal inflammation, thus alleviating the gastrointestinal toxicity of chemotherapy (Fig. 6). However, there were shortcomings in exploring *B.longum* SX-1326 for improvement of post-chemotherapy side effects, and a drug control group was not established to further compare the effectiveness of the drug with that of utilizing *B. longum* SX-1326 alone for improvement of nausea and vomiting and intestinal inflammation.

## Conclusion

Our results suggest that *B. longum* SX-1326 has great potential in the adjuvant irinotecan treatment of AOM/DSS-induced CRC mouse models. This is due to the activation of the p53 signaling pathway, which upregulates the expression of apoptotic proteins. In addition, our results showed that *B. longum* SX-1326 treatment alleviated post-chemotherapy nausea and vomiting by modulating the GBA and reducing the expression of neurotransmitters such as 5-HT in the brain tissue. It also down-regulates TLR4/MyD88/NF-κB signaling pathway and the expression of inflammatory factors in the intestine to restore the diversity of intestinal microbiota and improve diarrhea. It provides a new idea for clinical search of a safe and efficient adjuvant for CRC chemotherapy.

### Supplementary Information

**Additional file 1: Supplementary Material Table S1.**

**Additional file 2: Supplementary Material Table S1.**

**Additional file 3:  Supplementary Figures.**

## Data Availability

The raw reads were preserved in the Sequence Read Archive (SRA) database of NCBI. The accession numbers can be found below: PRJNA996752 (SRA).
