# Supplementary material for: Bifidobacterium longum SX-1326 ameliorates gastrointestinal toxicity after irinotecan chemotherapy via modulating the P53 signaling pathway and brain-gut axis
Source: BMC Microbiol. 2024 Jan 3;24:8. doi: 10.1186/s12866-023-03152-w (PMC10763180; doi:10.1186/s12866-023-03152-w)
Supplement: Supplementary file 1 — Additional file 1: Supplementary Material Table S1. [file 12866_2023_3152_MOESM1_ESM.docx]

**Supplementary Material Table S1**

| **qPCR target** | **Forward 5’-3’** | **Reverse5’-3’** |
| --- | --- | --- |
| **IL-1β** | GTGTCTTTCCCGTGGACCTTC | TCATCTCGGAGCCTGTAGTGC |
| **TNF-α** | GTGGAACTGGCAGAAGAGGCA | AGAGGGAGGCCATTTGGGAAC |
| **IL-6** | CTTCTTGGGACTGATGCTGGTGAC | AGGTCTGTTGGGAGTGGTATCCT |
| **GAPDH** | CTCGTGGAGTCTACTGGTGT | GTCATCATACTTGGCAGGTT |
