# Supplementary material for: Bifidobacterium longum SX-1326 ameliorates gastrointestinal toxicity after irinotecan chemotherapy via modulating the P53 signaling pathway and brain-gut axis
Source: BMC Microbiol. 2024 Jan 3;24:8. doi: 10.1186/s12866-023-03152-w (PMC10763180; doi:10.1186/s12866-023-03152-w)
Supplement: Supplementary file 2 — Additional file 2: Supplementary Material Table S1. [file 12866_2023_3152_MOESM2_ESM.docx]

**Supplementary Material Table S2**

| Antibodies | Source | Cat. No. |  |
| --- | --- | --- | --- |
| β-actin | Abcam | ab8226 |  |
| TLR4 | CST | 14358S |  |
| MyD88 | CST | 4283S |  |
| p-p65 | CST | 3033S |  |
| p65 | CST | 8242S |  |
| p-p53 | WanLei | WL02504 |  |
| p53 | Abcam | ab26 |  |
| Bax | CST | 2772S |  |
| Bcl-2 | proteintech | 12789-1-AP |  |
| Caspase-3 | CST | 9662S |  |
| Cleaved-Caspase-3 | CST | 9661S |  |
| COX-2 | CST | 12282S |  |
| Occludin | Proteintech | 66378-1-Ig |  |
| Claudin-1 | Abcam | ab180158 |  |
| goat rabbit antibody | Abcam | 205718 |  |
| goat anti-mouse | Abcam | 97265 |  |
| NK1R | Affinity | DF4996 |  |
| 5HT3R | Affinity | DF7437 |  |
| c-Fos | Servicebio | GB12069 |  |
