# Supplementary material for: Bifidobacterium longum SX-1326 ameliorates gastrointestinal toxicity after irinotecan chemotherapy via modulating the P53 signaling pathway and brain-gut axis
Source: BMC Microbiol. 2024 Jan 3;24:8. doi: 10.1186/s12866-023-03152-w (PMC10763180; doi:10.1186/s12866-023-03152-w)

Figure 2B:

Bax（20KD）

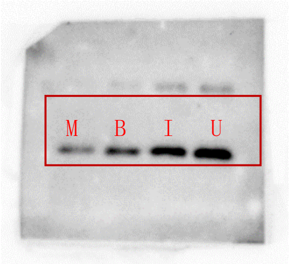

Figure 2B:

p53（53KD）

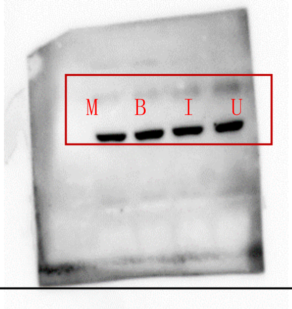

Figure 2B:

p-p53(53KD)

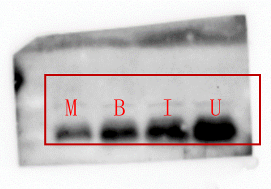

Figure 2B:

Bcl-2(26KD)

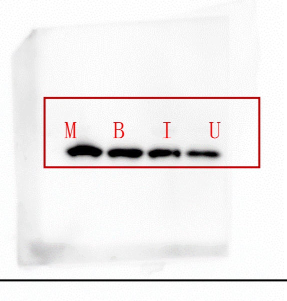

Figure 2B:

β-actin(42KD)

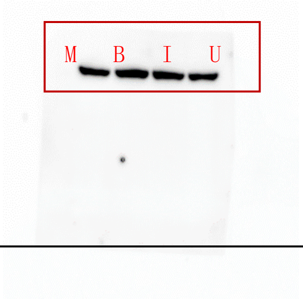

Figure 2E:

Caspase-3(32KD)

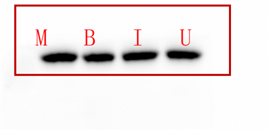

Figure 2E:

Cleaved Caspase-3(19KD)

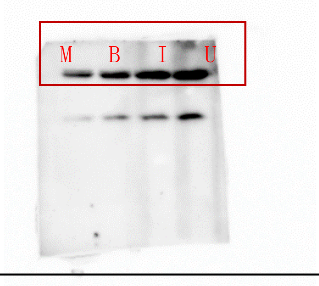

Figure 5E:

MyD88(33KD)

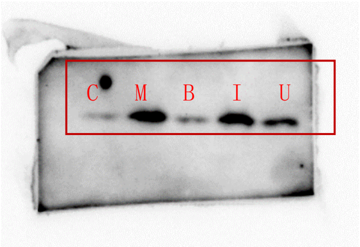

Figure 5E:

p65(65KD)

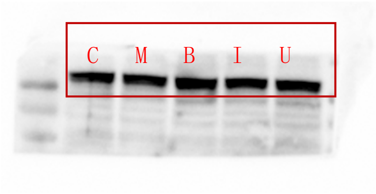

Figure 5E:

p-p65(65KD)

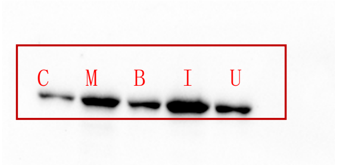

Figure 5E:

TLR4(90KD)

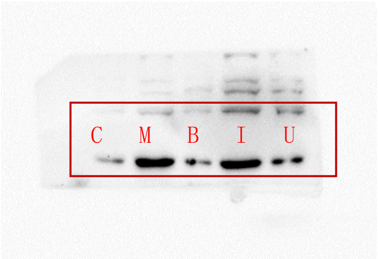

Figure 5E:

β-actin(42KD)

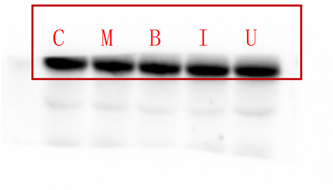

Figure 5I:

COX-2(72KD)

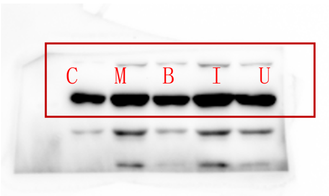

Figure 5I:

β-actin(42KD)

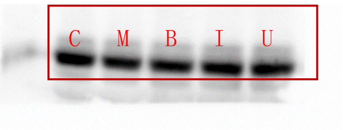

Figure 5k:

Claudin-1(20KD)

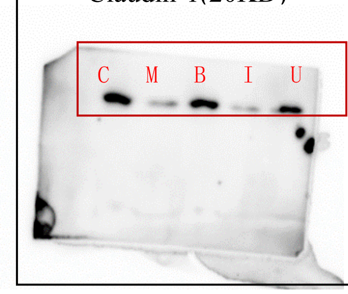

Figure 5k:

Occludin(65KD)

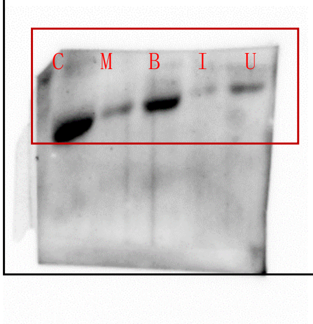

Figure 5k:

β-actin(42KD)

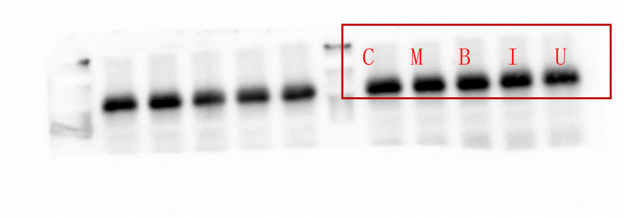

Supplement: Supplementary file 3 — Additional file 3: Supplementary Figures. [file 12866_2023_3152_MOESM3_ESM.pdf]
